# Connexin 43 and Sonic Hedgehog Pathway Interplay in Glioblastoma Cell Proliferation and Migration

**DOI:** 10.3390/biology10080767

**Published:** 2021-08-12

**Authors:** Filippo Torrisi, Cristiana Alberghina, Debora Lo Furno, Agata Zappalà, Samuel Valable, Giovanni Li Volti, Daniele Tibullo, Nunzio Vicario, Rosalba Parenti

**Affiliations:** 1 Section of Physiology, Department of Biomedical and Biotechnological Sciences, Section of Physiology, University of Catania, 95123 Catania, Italy; filippo.torrisi@unict.it (F.T.); cristiana.alberghina@phd.unict.it (C.A.); lofurno@unict.it (D.L.F.); azappala@unict.it (A.Z.); 2 GIP Cyceron, ISTCT/CERVOxy Group, CEA, CNRS, Normandie Université, UNICAEN, 14074 Caen, France; samuel.valable@cnrs.fr; 3 Section of Biochemistry, Department of Biomedical and Biotechnological Sciences, Section of Physiology, University of Catania, 95123 Catania, Italy; livolti@unict.it (G.L.V.); d.tibullo@unict.it (D.T.)

**Keywords:** GBM, connexin, gap junction, smoothened, GLI1

## Abstract

**Simple Summary:**

Glioblastoma is the product of accumulated genetic and epigenetic alteration where tumor cells support each other through cellular communication mechanisms and deregulated signalling processes. The autocrine and paracrine pathways between the intracellular and extracellular milieu is mediated by connexin 43, the main gap junction-forming protein driving glioblastoma progression. In this scenario, sonic hedgehog pathway, a key deregulated pathway involved in cell network signalling may affect connexin 43 expression, promoting glioblastoma pathobiology. In this study, we sought to explore how the modulation of the sonic hedgehog affects connexin 43 inducing glioblastoma hallmarks. To do this we evaluated biological effects of sonic hedgehog pathway modulation by purmorphamine and cyclopamine, a smoothened agonist and antagonist, respectively. We revealed that cell migration and proliferation are associated with connexin 43 expression upon sonic hedgehog modulation. Our study suggests that sonic hedgehog and connexin 43 axis may represent a potential therapeutic strategy for glioblastoma.

**Abstract:**

Glioblastoma (GBM) represents the most common primary brain tumor within the adult population. Current therapeutic options are still limited by high rate of recurrences and signalling axes that promote GBM aggressiveness. The contribution of gap junctions (GJs) to tumor growth and progression has been proven by experimental evidence. Concomitantly, tumor microenvironment has received increasing interest as a critical process in dysregulation and homeostatic escape, finding a close link between molecular mechanisms involved in connexin 43 (CX43)-based intercellular communication and tumorigenesis. Moreover, evidence has come to suggest a crucial role of sonic hedgehog (SHH) signalling pathway in GBM proliferation, cell fate and differentiation. Herein, we used two human GBM cell lines, modulating SHH signalling and CX43-based intercellular communication in in vitro models using proliferation and migration assays. Our evidence suggests that modulation of the SHH effector smoothened (SMO), by using a known agonist (i.e., purmorphamine) and a known antagonist (i.e., cyclopamine), affects the CX43 expression levels and therefore the related functions. Moreover, SMO activation also increased cell proliferation and migration. Importantly, inhibition of CX43 channels was able to prevent SMO-induced effects. SHH pathway and CX43 interplay acts inducing tumorigenic program and supporting cell migration, likely representing druggable targets to develop new therapeutic strategies for GBM.

## 1. Introduction

Despite the multimodal and synergistic approaches, combining surgical, pharmacological and radiotherapeutic strategies, glioblastoma (GBM) is still the most lethal brain tumor, characterized by high rate of recurrences and a dismal prognosis [1,2]. Emerging research in the field, has led to the investigation of molecular pathogenesis in order to find the biological mechanisms underlying GBM growth, aggressiveness and resistance [3,4,5,6,7].

Sonic Hedgehog (SHH) represents one of the most relevant signalling pathways mediating both cell fate and differentiation [8,9,10]. The activation of 7-transmembrane protein Smoothened (SMO) determines the GLI-Kruppel family member 1 (GLI1) nuclear translocation promoting proliferation, stem cell renewal and survival [10,11,12]. SHH dysregulation has been reported in brain tumors, including GBM [13,14,15]. Indeed, proliferation and self-renewal of GBM stem cells (GSCs) are regulated by SHH signalling activation [16,17], whereas their inhibition have been associated to an increased chemotherapy response, reducing of GSCs resistance and maintenance [18,19,20]. Moreover, infiltrative growth of GBM has been associated to the aberrant activation of SHH pathway by the enhancement of migration ability [21]. SHH pathway is also a key component of the autocrine and paracrine signalling promoting tumor progression, due to uncontrolled proliferation, sustained angiogenesis and invasiveness [22].

Intercellular communication in GBM represents an active research field. Indeed, cell network and autocrine/paracrine signalling were found to modulate the molecular mechanisms of GBM proliferation [23]. In particular, cell-to-cell and cell-to-extracellular fluid communication stimulate GBM cells migration, enhancing the infiltrative pattern, preluding to therapeutic failure and inevitable recurrences [24,25].

Connexins (Cxs) are integral membrane proteins that assemble to form gap junctions (GJs), mediating a direct cytoplasmic connection between adjacent cells, and hemichannels (HCs), providing autocrine and paracrine pathways between the intracellular and extracellular milieu [26]. Allowing exchanges of ions, metabolites, second messengers and small molecules (less than 1000 kDa), GJs and HCs represent key cellular substrates of many significant biological processes throughout life in both physiological and pathological conditions [27,28,29]. Particularly, GJs- and HCs-mediated intercellular crosstalk represents an undoubted way through which different cell types regulate tumor development and progression. Controlling Cxs expression and activity implies significant changes in microenvironment composition and intercellular signalling, a major contributor in tumor cell stimulation and stress resistance.

Among the most important Cxs involved in tumor trophism, connexin 43 (CX43) implication is supported by a plethora of data, describing CX43-based channels as major microenvironmental conditioning mediators [26,28,30]. It has been proposed that CX43 regulates the expression levels of proteins involved in cell growth independently by their channel forming properties, given the multifaceted role of CX43 carboxyl tail, which exerts a number of effects ranging from controlling the translocation of transcription factor regulators into the nucleus to the enhancing of the migration of glioma cells out of the tumor core by interacting with cytoskeleton elements [31].

The close relationship between CX43 and SHH consistently stands out in several scenarios. It appears primarily during embryonic development, when direct cell-to-cell communication is the key mechanism for structures patterning; in this context, for instance, synergistic SHH signalling and CX43 expression, establishing GJs networks synchronizing the Ca^2+^ profile among cells, coordinate collective cell movements, strictly dependent on SHH signalling activation [32]. This phenomenon also occurs in tumor-associated conditions characterized by cell invasion, further confirming a mechanistic link between development and tumorigenesis [33].

Based on such evidence, we hypothesized that a close association between CX43 and SHH pathway in promoting cell network, cell renewal and tumor microenvironment may be critical to sustain the GBM malignancy progression.

For this purpose, we first evaluated in vitro the metabolic and cytotoxic effect of purmorphamine and cyclopamine, a SMO agonist and antagonist, respectively [34,35], and the biological effect of SHH pathway modulation with particular regard to the expression of CX43 and cell migration.

## 2. Materials and Methods

### 2.1. Cell Lines Culture and SMO Modulation

Experiments were performed using U-251 MG and T98-G human glioblastoma cell lines. Cells were purchased from European Collection of Authenticated Cell Cultures (ECACC, Public Health England, Porton Down Salisbury, Wiltshire, UK) and cultured in DMEM high glucose supplemented with 10% Serum Fetal Bovin (FBS), 100 IU/mL penicillin (Penicillin-Streptomycin solution) and 2 mmol/L glutamine. Cells were maintained in an exponentially growing culture condition in an incubator at 37 °C in a humidified atmosphere (95% air and 5% CO_2_) and were routinely sub-cultured in standard tissue culture flasks. All experiments employed cells at a passage *n* < 25.

Purmorphamine (Cat#72204, Stem Cell Technologies), cyclopamine (Cat#S1146, Selleckchem, Rome, Italy) and ioxynil octanoate (Cat#33381-100MG, Merk, Milan, Italy) were prepared as a stock solution at 10 mM and stored at −20 °C. For cells treatment, drugs were diluted at the final concentration in culture medium not exceeding 0.5% of Dimethyl sulfoxide (DMSO, Merk, Milan, Italy) in order to not affect cell viability. Untreated cells were supplemented with DMSO, as vehicle.

### 2.2. Rate of Cell Growth Assay

The rate of cell growth (R.C.G.) was calculated as previously described [11]. Briefly, cells were seeded on six multiwell plates at a final density of 2 × 10^4^ cells/cm^2^. Cells were counted and seeded at the same density every 2 or 3 days for a total of five consecutive passages. The R.C.G. was calculated by counting the number of viable cells by trypan blue vital stain exclusion and dividing it by the number of plated cells; ratios were divided by the number of days per passage.

### 2.3. Immunofluorescence

Immunofluorescence analysis on GBM cell lines was performed as previously described [36]. Briefly, cells were seeded in cover glass placed into multiwell 24 plates at final density of 2 × 10^4^ cells/cm^2^. Cells were fixed in 4% paraformaldehyde (PFA) at room temperature for 10 min. Then, cells were incubated with blocking solution (10% normal goat serum, NGS, in PBS) for 1 h at room temperature. Samples were then incubated overnight at 4 °C with the following primary antibodies diluted in incubating solution (1% NGS in PBS): mouse anti-GFAP (1:500, Cat#MAB16117, RRID: N/A, Immunological Sciences, Rome, Italy); rabbit anti-CX43 (1:200, Cat#3512S, RRID: AB_2294590, Cell Signalling, Danvers, MA, USA); rabbit anti-SMO (1:1000, Cat# ab72130, RRID: AB_1270802, Abcam, Cambridge, UK), rabbit anti-Ki-67 (1:200, Cat#ab15580, RRID: AB_443209, Abcam, Cambridge, UK). Then, after three washes in PBS, samples were incubated for 1 h at room temperature with the appropriate combination fluorescence goat secondary antibodies: Goat anti-mouse Alexa Fluor 488 (1:1000, Cat# A-11001, RRID: AB_2534069, Thermo Fisher Scientific); Goat anti-mouse, Alexa Fluor 546 (1:1000, Cat# A-11003, RRID: AB_2534071, Thermo Fisher Scientific); Goat anti-rabbit, Alexa Fluor 488 (1:1000, Cat# A27034, RRID: AB_2536097, Thermo Fisher Scientific); Goat anti-rabbit, Alexa Fluor 546 (1:1000, Cat# A11010, RRID: AB_143156, Invitrogen, Waltham, MA, USA). Nuclei were counterstained with 4′,6-diamidino-2-phenylindole (Dapi, 1:1000, Cat# D1306, Invitrogen) for 5 min at room temperature. Slides were mounted with fluorescent mounting medium Permafluor (ThermoScientific) and digital images were acquired using a Leica DM IRB (Leica Microsystems, Buccinasco, Milano, Italy) fluorescence microscope or the Leica TCS SP8 confocal microscope. In order to quantify the fluorescence intensity of CX43, *n* = 4 representative regions of interest (ROIs) were quantified by application of a Isodata threshold using ImageJ analysis software. Data were normalized over ROI total area and expressed as mean fluorescence intensity fold change (FC) over control ± SEM. CX43 frequency distribution was calculated on ROI based on GFAP immunofluorescence signal and the mean fluorescence intensity of CX43 was divided by the ROI area. Data are shown as violin plots of this ratio for *n* ≥ 20 cells per group.

### 2.4. Cytotoxicity and Metabolic Turnover Assays

To assess cytotoxicity and metabolic turnover, we used Lactate dehydrogenase (LDH) assay (i.e., cytotoxicity) or 3-(4,5-dimethylthiazol-2-yl)-2,5-diphenyltetrazolium bromide (MTT) assay (i.e., metabolic turnover) as previously described [28,37], with minor modifications. Cells were distributed in 96-well plates (Costar, Milan, Italy) at a final density of 10,000 cells/well/100 µL and incubated for 24 h. The day after, cells were exposed to drugs as above described, and incubated for 4, 24 and 48 h. On the day of each time point, medium was removed and processed as manufacturer’s instructions for the LDH-viability assay (CytoSelectTM LDH cytotoxicity assay kit, Cell Biolabs, Milan, Italy). For metabolic turnover, MTT at a final concentration of 1 mg/mL was added to each well and incubated for 3 h under standard culture conditions. Media were then gently removed, 200 µL of MTT solvent (DMSO) was added, and cells were stirred on an orbital shaker for 10 min at room temperature. The absorbance was measured using a Varioskan Flash spectrophotometer (Thermo Scientific, Milan, Italy) at 570 nm. Data were expressed as the percentage of MTT reduction versus control cells. Each experiment was performed three times with six replicates per condition during each experimental run.

### 2.5. Clonogenic Assay

Clonogenic assay was performed as previously described [38]. Briefly, U-251 MG and T98-G cells were plated at 50/cm^2^ cells per well in triplicate, and were incubated for 8 h, allowing them to attach on the well plate. Then, cells were treated with either vehicle, purmorphamine or cyclopamine at a final concentration of 0.1 µM, 1 µM and 10 µM. Each plate was incubated for 7–10 days, and after colonies were formed, they were fixed with 4% PFA for 15 min and stained with 1% crystal violet for 30 min at room temperature. Colonies which accounted for more than 50 cells were considered as clones. Each assay was repeated in triplicate in three independent experiments. Surviving fractions were calculated as the ratio of colonies counted over the cell plated multiplied for the plating efficiency of the vehicle, according to the protocol of Franken et al. [39].

### 2.6. Immunoblotting

For Western blot analysis, cells were seeded in six multiwell plates at final density of 3 × 10^6^/well and incubated at 37 °C before drug exposure. Drugs were added at the final concentration of 1 µM on cells and maintained for 24 h. Then, cells were washed in PBS, detached by scraper and centrifuged for 5 min at 1200 rpm to collect dry pellet, that were stored at −80 °C until use. Proteins were extracted using RIPA Lysis Buffer (50 μL/sample; Abcam, Cambridge, UK) supplemented with protease inhibitor (1:100, Cat# P8340, Merk, Milan, Italy). Briefly, samples were incubated for 20 min at room temperature and centrifuged at 13,000× *g* for 3 min. An equal amount of proteins (30 μg) were electrophoresed on 4−20% SDS-PAGE gels and transferred to nitrocellulose membranes as previously described [40,41]. Membranes were incubated for 1 h at room temperature with blocking buffer (5% non-fat milk in 0.1% tween-20 in PBS) and then overnight at 4 °C with primary antibodies diluted in blocking buffer. The following primary antibodies were used for immunoblotting: Rabbit anti-GLI1 (1:1000, Cat# ab49314, RRID: AB_880198, Abcam, Cambridge, UK), Rabbit anti-CX43 (1:1000, Cat# C6219, RRID: AB_476857, Merk, Milan, Italy), and mouse anti-GAPDH (1:1000, Cat# ab181602, RRID: AB_2630358, Abcam, Cambridge, UK). Then, membranes were washed three times in 0.1% tween-20 in PBS and then incubated for 1 hr at room temperature with the appropriate secondary antibody: IRDye 800CW Goat anti-mouse (1:5000, Cat# 925-32210: RRID: AB_2687825, LI-COR Biosciences) or goat anti-rabbit (IRDye 680RD; LI-COR Biosciences, Cat# 926-68071, RRID: AB_2721181, 1:10,000). Proteins bands were imaged using an Odyssey Infrared Imaging Scanner (LI-COR Biosciences, Milan, Italy) and protein levels were quantified by densitometric analysis. The density of each band was quantified using ImageJ analysis software and the band density was normalized to the GAPDH optical density measured in the same membrane. For immunoblotting quantification, the density of each band was quantified using ImageJ analysis software and band density was normalized to the GAPDH optical density measured in the same membrane. All values are shown as the mean fold change (FC) over control ± SEM.

### 2.7. Migration Assay

U-251 MG and T98-G cells were seeded in 24-well plates at a final concentration of 3 × 10^5^/well and grown until cells reached about 90% confluency. The day after a central and linear scratch was created using a 200 μL tips and samples were washed with PBS to remove residuals cells; media were then replaced with migration medium (DMEM high glucose, 100 IU/mL Penicillin-Streptomycin and 2 mmol/L glutamine) containing vehicle, 1 µM purmorphamine and/or 1 µM cyclopamine added alone or in combination with ioxynil octanoate at a final concentration of 10 µM. For the quantification of migration index, the total scratch area was segmented from one edge to the opposite edge and the total size of the wound was then measured at 0 and 24 h post scratch and migration index was calculated as percentage ratio of scratch area at 0 h -scratch area at 24 h over scratch area at 0 hrs. All values are shown as the mean FC over 0 hrs ± SEM.

### 2.8. Statistical Analysis

All tests were performed in GraphPad Prism (version 5.00, GraphPad Software, San Diego, CA, USA). Data were tested for normality using a D’Agostino and Pearson omnibus normality test and subsequently assessed for homogeneity of variance. For multiple comparison, one-way ANOVA was used where appropriate, followed by Holm–Šídák post-hoc test.

## 3. Results

### 3.1. Human GBM Cell Lines Actively Express SMO

**Figure 1:**
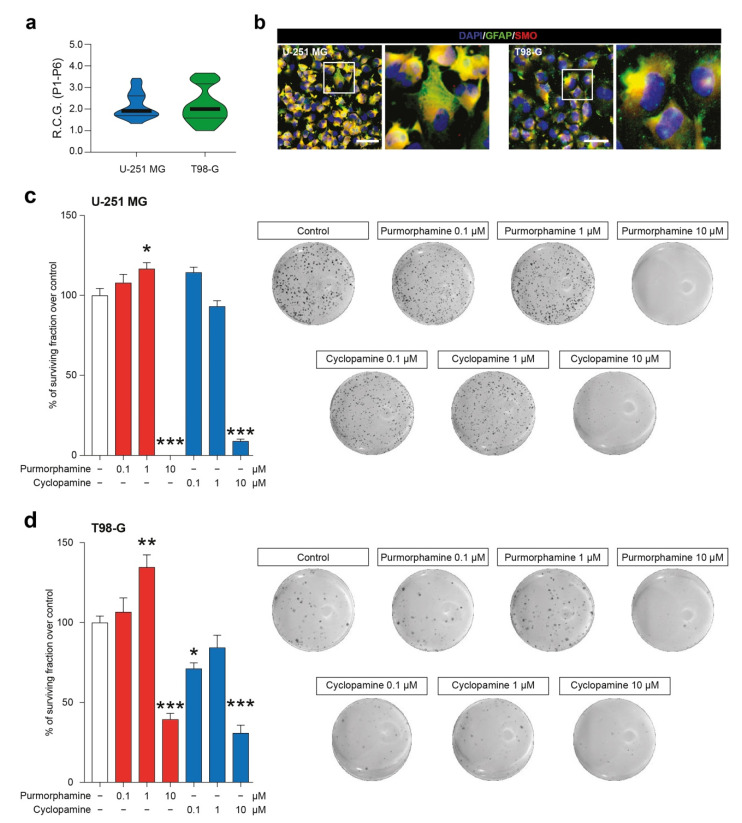
SMO modulation in U-251 MG and T98-G cells impacts on surviving fraction. (**a**) Rate of cell growth of human U-251 MG and T98-G cell lines, data are shown as violin plot of R.C.G. of *n* = 3 replicates over *n* = 6 passages. (**b**) Representative microphotographs of human U-251 MG andT98-G cells expressing GFAP and SMO; nuclei were counterstained with DAPI; scale bar = 20 μm. (**c**,**d**) Surviving fraction and representative wells of U-251 MG (**c**) and T98-G cell lines (**d**) exposed to increasing concentration of purmorphamine and cyclopamine (0.1–10 µM). Data are expressed as mean ± SEM of *n* = 3 independent experiments; * *p*-value < 0.05, ** *p*-value < 0.01 and *** *p*-value < 0.001 versus control cell cultures treated with vehicle; one-way ANOVA with Holm–Šídák post-hoc test. R.C.G.: rate of cell growth. P: passage.

We first characterized two GBM-derived cell lines, studying their stability over passages in vitro (Supplementary Figure S1) and their R.C.G. (Figure 1a). Our data showed that U-251 MG and T98-G displayed a similar profile in terms of total number of cells over passages (P1–P6) and that no statistical differences were observed in R.C.G. (respectively: 2.15 ± 0.10 vs. 2.17 ± 0.14 for U-251 MG and T98-G). We further expand our characterization to study the potential of our cell lines to respond to known modulators of SHH signalling pathway acting on SMO. To do that, we performed an immunofluorescence analysis for GFAP and SMO, confirming the astroglial origin of analyzed cells and the presence of SMO on all tested cell lines (Figure 1b). This evidence indicated that U-251 MG and T98-G expressed the druggable target SMO.

### 3.2. Modulation of SHH Signalling Pathway Impact Cytotoxicity, Metabolic Turnover and Cell Proliferation

Given the presence of SMO, we decided to test cytotoxicity at 4, 24 and 48 h on human GBM cell lines exposed to SMO modulators using a known agonist (i.e., purmorphamine) and a known antagonist (i.e., cyclopamine) at concentration ranging from 0.01 to 10 µM. We performed a lactate dehydrogenase assay showing no significant effects in U-251 MG and T98-G as compared to vehicle treated controls (Supplementary Figure S2). These results showed that, in our experimental conditions, neither purmorphamine nor cyclopamine exerted significant cytotoxic effects on GBM cell lines as a single dose of 0.01 to 10 µM.

In order to evaluate a potential impact of SMO modulation on metabolic and mitochondrial function, we performed an MTT turnover assay. Notably, in U-251 MG cells a statistically significant increase in MTT turnover was observed as soon as 4 h post-exposition in cultures treated with 10 µM of purmorphamine (129.6 ± 5.2% purmorphamine 10 µM; *p*-value < 0.05; Supplementary Figure S3). This effect was also present at 24 h with both 1 µM and 10 µM of purmorphamine treated cultures (135.6 ± 6.7% purmorphamine 1 µM and 132.5 ± 12.3% purmorphamine 10 µM; *p*-value < 0.05; Supplementary Figure S3). In T98-G cell line we were not able to observe any significant effect on MTT turnover at 4 h post-exposition with purmorphamine. Interestingly, at 24 h T98-G cells exposed to purmorphamine significantly increase their MTT turnover (138 ± 7.7% purmorphamine 10 µM; *p*-value < 0.01; Supplementary Figure S3). This effect was retained for 48 hrs post purmorphamine exposition (136 ± 9.1% purmorphamine 10 µM; *p*-value < 0.01; Supplementary Figure S3). In addition, in T98-G, but not in U-251 MG, a transient but significant reduction of MTT-turnover at 4 hrs after 10 µM cyclopamine exposition was observed (34.9 ± 1.7% cyclopamine 10 µM; *p*-value < 0.001; Supplementary Figure S3).

These evidence prompted us to perform clonogenic assay in order to evaluate the long-term effects of SHH pathway modulation in colonies formation and, particularly, the ability of cyclopamine to reduce cell proliferation. The colonies formation assay revealed that both cell lines were significantly impacted by 1 µM purmorphamine as compared to the untreated cells (117 ± 3.8%; *p*-value < 0.05 vs. 100 ± 3.8% vehicle for U-251 MG; 135 ± 7.7%; *p*-value < 0.01 vs. vehicle 100 ± 4.1% for T98-G; Figure 1c,d). This concentration was able to increase the % of surviving fraction, thus supporting the idea that SHH pathway foster GBM proliferation. Of note, despite LDH and MTT data, clonogenic assay on U-251 MG and T98-G cell lines revealed that long-term exposition to 10 µM of purmorphamine strikingly reduced the % of surviving fraction (Figure 1c,d). Of note, a reduction of surviving fraction was observed in both cell lines exposed to increasing concentration of cyclopamine, in particular with 10 µM of drug (Figure 1c,d).

### 3.3. SHH Pathway Activation Is Related to CX43 and Ki-67 in Human GBM Cell Lines

**Figure 2:**
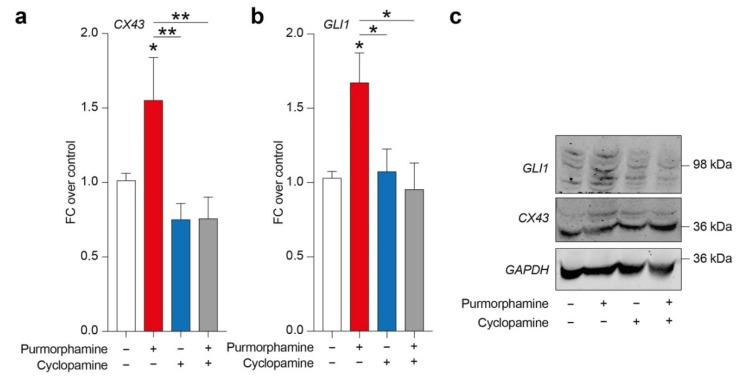
SHH pathway activation is related to CX43 increased expression levels in U-251 MG. (**a**–**c**) CX43 and GLI1 expression levels (**a**,**b**) and representative blots (**c**) on human U-251 MG cells exposed to 1 µM of purmorphamine and/or 1 µM cyclopamine. Data are mean FC over control ± SEM of *n* = 3 independent experiments; * *p*-value < 0.05, ** *p*-value < 0.01 versus control or between groups; one-way ANOVA with Holm–Šídák post-hoc test. FC: fold change.

In order to evaluate a potential relation between SHH signalling pathway and CX43, we tested purmorphamine, cyclopamine and combination of these drugs in inducing CX43 expression levels and GLI1, a main intracellular effector of canonical SHH signalling pathway. Analysis of protein expression levels revealed a significant increase in both CX43 and GLI1 in cells exposed to 1 µM of purmorphamine, and this effect was not observed in cyclopamine- or purmorphamine + cyclopamine-treated cells (Figure 2a–c).

**Figure 3:**
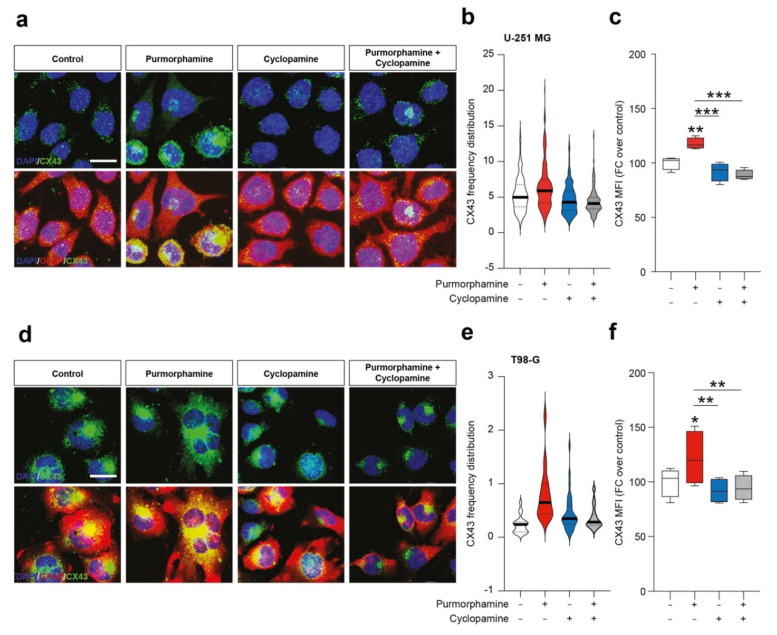
SHH pathway activation increase immunofluorescence intensity of CX43 in human GBM cell lines. (**a**–**c**) Representative microphotographs of CX43 and GFAP (**a**), CX43 frequency distribution (**b**) and quantification of CX43 MFI (**c**) of control human U-251 MG cells and U-251 MG cells exposed to 1 µM of purmorphamine and/or cyclopamine. (**d**–**f**) Representative pictures of CX43 and GFAP (**d**), CX43 frequency distribution (**e**) and quantification of CX43 MFI (**f**) of control human T98-G cells and T98-G cells exposed to 1 µM of purmorphamine and/or cyclopamine. Data in (**b**,**e**) are shown as violin plot of *n* ≥ 20 cells and data in (**c**,**f**) are shown via standard box and whiskers plot of *n* = 4 independent experiments; * *p*-value < 0.05, ** *p*-value < 0.01, *** *p*-value < 0.001 versus control or between groups; one-way ANOVA with Holm–Šídák post-hoc test. In (**a**,**d**) nuclei were counterstained with DAPI; scale bar in (**a**,**d**) = 10 μm. MFI.

To further expand this evidence, we performed an immunofluorescence analysis on U-251 MG and T98-G cell lines for CX43 and GFAP. We found purmorphamine induced an overall increase of CX43 Mean Fluorescence Intensity (MFI) in U-251 MG (Figure 3a–c) and that this phenomenon was particularly pronounced in some of the cells that expressed particularly high levels of CX43, while GFAP immunopositivity was comparable among cells (Figure 3a). We observed similar phenomena in T98-G cells, even if results showed high levels of CX43 also in control condition (Figure 3d). Additionally in this cell line, purmorphamine significantly increased CX43 MFI and this effect was reverted by co-treatment with cyclopamine (Figure 3e–f).

**Figure 4:**
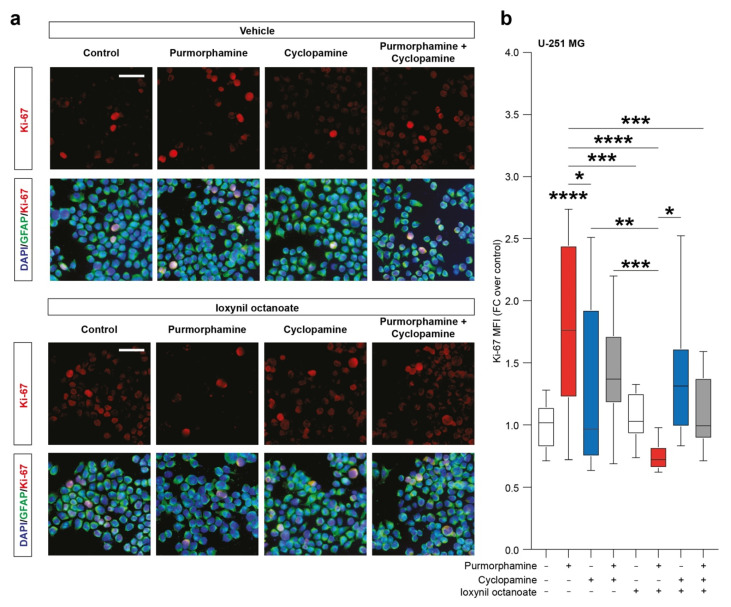
SHH pathway activation increase Ki-67 in U-251 MG cell line. (**a**,**b**) Representative microphotographs of Ki-67 and GFAP immunofluorescence analysis (**a**) and quantification of Ki-67 MFI (**b**) of control U-251 MG cells and U-251 MG cells exposed to 1 µM of purmorphamine and/or cyclopamine, treated with either vehicle and ioxynil octanoate; nuclei were counterstained with DAPI; scale bar = 20 μm. Data are mean ± SEM of *n* = 3 independent experiments; * *p*-value < 0.05 and ** *p*-value < 0.01, *** *p*-value < 0.001, **** *p*-value < 0.0001 versus control or between groups; one-way ANOVA with Holm–Šídák post-hoc test. MFI.

To evaluate the effects of SHH signalling pathway modulation on the proportion of proliferating cells we then performed an immunofluorescence analysis of the proportion of Ki-67 positive cells. Our analysis pointed out a significant increase in the nuclear Ki-67 MFI in purmorphamine treated cells (1.77 ± 0.2 purmorphamine versus 1.0 ± 0.04 control, *p*-value < 0.0001, Figure 4a,b) and this effect was not observed in cultures cotreated with cyclopamine (1.45 ± 0.1 cyclopamine, Figure 4a,b). Interestingly, in U-251 MG cultures exposed to purmorphamine and treated with ioxynil octanoate, a selective inhibitor of CX43-based GJs, the nuclear Ki-67 MFI was not increased versus control nor versus ioxynil octanoate-treated cells (0.75 ± 0.02 purmorphamine + ioxynil octanoate versus 1.1 ± 0.04 control ioxynil octanoate, Figure 4a,b). Of note, cyclopamine and purmorphamine + cyclopamine cotreatment cells treated with ioxynil octanoate showed not significant changes in Ki-67 MFI (1.38 ± 0.1 cyclopamine + ioxynil octanoate and 1.11 ± 0.1 purmorphamine + cyclopamine + ioxynil octanoate, Figure 4a,b).

### 3.4. SHH-CX43 Axis Induces Migration Enhancement in Human GBM Cell Lines

**Figure 5:**
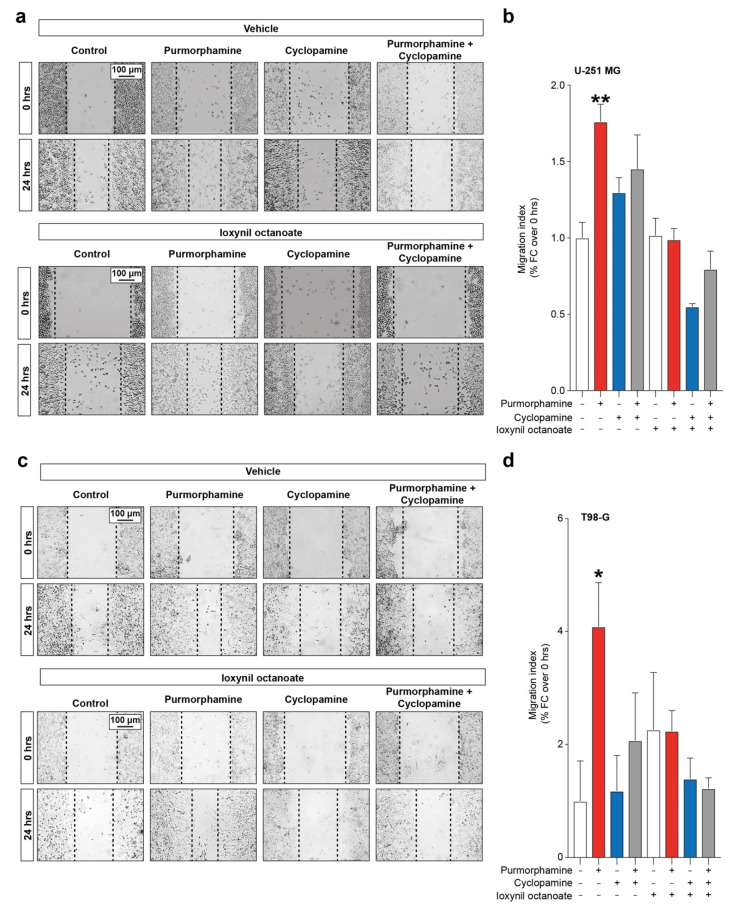
SHH-CX43 axis induces migration enhancement in human GBM cell line. (**a**,**b**) Representative images (**a**) and migration index (**b**) of control human U-251 MG cells and U-251 MG exposed to 1 µM of purmorphamine and/or cyclopamine, treated with either vehicle or ioxynil octanoate. (**c**,**d**) Representative images (**c**) and migration index (**d**) of control human T98-G cells and T98-G exposed to 1 µM of purmorphamine and/or cyclopamine, treated with either vehicle or ioxynil octanoate. Data are shown as mean fold change over 0 hrs ± SEM of n = 3 independent experiments; * *p*-value < 0.05 and ** *p*-value < 0.01 versus control; one-way ANOVA with Holm–Šídák post-hoc test.

Given the importance of both SHH signalling pathway and CX43-based channels in modulating proliferation and cell migration, we moved to investigate the interplay between these factors in influencing GBM cell lines migration. Our data showed a significant increase in migration index in U-251 MG cells exposed to purmorphamine 1 µM (1.76 ± 0.11 purmorphamine versus 1.00 ± 0.10 control, *p*-value = 0.0083, Figure 5a,b), which was not observed in cyclopamine cotreated cells (1.45 ± 0.22 purmorphamine + cyclopamine, Figure 5a,b). Such an effect was not observed in cell cultures exposed to ioxynil octanoate, that abolish purmorphamine-induced migration increase (1.02 ± 0.11 control + ioxynil octanoate, 0.99 ± 0.08 purmorphamine + ioxynil octanoate 0.55 ± 0.02 cyclopamine + ioxynil octanoate, 0.80 ± 0.12 purmorphamine + cyclopamine + ioxynil octanoate, Figure 5a,b). These results were also confirmed in T98-G cells lines, in which we observed a significant increase in migration index upon purmorphamine exposition (4.09 ± 0.78 purmorphamine versus 1.00 ± 0.71 control, *p*-value = 0.03, Figure 5c,d) that was abolished by cyclopamine (1.18 ± 0.63 cyclopamine, Figure 5c,d). Moreover, in this cell line no significant changes were observed in cultures exposed to ioxynil octanoate (2.26 ± 1.01 control + ioxynil octanoate, 2.24 ± 0.36 purmorphamine + ioxynil octanoate, 1.39 ± 0.37 cyclopamine + ioxynil octanoate and 1.23 ± 0.19 purmorphamine + cyclopamine + ioxynil octanoate, *p*-values > 0.05 for all comparisons, Figure 5c,d).

## 4. Discussion

GBM, a WHO grade IV glioma, represents the most common primary brain tumor within the adult population. There is an urgent need to develop novel therapeutic approaches to reduce overall morbidity, mortality and short-term and long-term adverse effects of current therapeutic approaches [42]. The path towards the development of druggable targets and effective therapeutic approaches is particularly challenging due to the infiltrative nature of this malignant glioma and its marked heterogeneity with the warring presence of self-renewing cancer stem cells. Therefore, a valid approach is represented by an in-depth knowledge of the mechanisms and players controlling its complex tumor microenvironment.

SHH signalling pathway is a crucial player not only in cell proliferation, self-renewal and differentiation modulation during central nervous system development and patterning [8], but also contributing to the development of various malignancies, including GBM [18,22]. It has been suggested that SHH signalling pathway promotes GBM-cell migration and invasion by increasing matrix metalloproteinase 2 (MMP2) and matrix metalloproteinase 9 (MMP9) production via the PI3K/AKT pathway [43,44] and by regulating the stem cell fraction in GBM cell lines [45]. The invasive behaviour of glioma cells induced by CX43-dependent signalling has been previously described by studies reporting that CX43 increases the secretion and activation of proteins associated with cell motility and extracellular matrix remodelling, also due to the tumorigenicity activation of neural progenitor spheroids and glioblastoma stem cells [46,47,48]. Indeed, inhibitors of the SHH pathway effector SMO have been successfully tested in both in vitro and in vivo GBM models and demonstrated to efficiently counteract self-renewal and tumor progression [20,45]. Another noteworthy factor is that SHH-GLI signalling has been associated to proliferation, survival, self-renewal and tumorigenicity of cancer stem cells with several markers of stemness differently characterizing the grade, growth and survival of pathology, [49,50,51], so that pharmacological modulation of SHH pathway has been proposed in different therapeutic plans to prevent tumor proliferation and recurrence [52].

A large amount of data supports the hypothesis that GJs- and HCs-mediated intercellular communication, by regulating the exchanges between adjacent cells and conditioning extracellular environment, also modulates survival, development and progression of tumor microenvironment as much as channel-independent mechanisms via the Cxs relationships with multiprotein complexes and pathways. The significant role exerted by GJs in GBM invasiveness and progression is not surprising since the condition in which a tumor microenvironment develops, is determined by the altered activity of GJs and HCs among the various cells, including endothelial cells, astrocyte, pericytes and neurons, coexisting in the so-called neurovascular unit (NVU) [53]. The dysregulation of homeostatic NVU microenvironment dramatically affect blood brain barrier (BBB) function, resulting in increased peripheral derived detrimental stimulation and peritumoral vasculature, finally inducing migration of GBM cells. In particular, CX43 proved to be an attractive target for GBM since it is dynamically expressed by highly invasive glioblastoma cells, showing a multifaceted appearance dependent on both GBM growth level and malignant phase so that some tumor cells would be expected to migrate (CX43 expressing cells) and others to proliferate (CX43 non-expressing cells) [31]. Indeed, alongside the traditional role, it needs to be considered the effect of Cxs independent of GJs and HCs but correlated to the close collaboration with partners of different signalling pathways, or gene expression regulation, involved in cellular transformation processes [54,55].

Over the years, increasing interests have been placed in clarifying the crosstalk between GJs- and HCs-mediated communication and SHH pathway [33]. A finely regulated relationship between SHH and CX43 has been suggested during developmental processes. Decreased or absent gap junctional coupling in Cx43 mouse mutants leads to altered expression of morphogens including SHH conducing to different phenotypic abnormalities [56,57].

Similarly, morphogenic factors including WNT and SHH play important roles in microglia/astrocytes glioma crosstalk, recapitulating developmental programs of the tissues and organs during early embryogenesis [16,58]. It has been documented that the plasticity of tumor progression critically depends on reciprocal interaction of tumor cells with the different players of microenvironment including connexin, morphogens and cytoskeleton elements which participate dynamically to malignant transformation [59].

Growth factors, cytokines and matrix proteins, released from tumor cells and host stromal cells, through different subsets of cellular interactions, give the tumor microenvironment the identity of a dynamic niche for tissue remodelling, where glioma cells can hijack the molecular programs involved in normal tissue development, including immune signalling pathways, to promote their own survival and expansion [60]. Moreover, it has been shown that SHH in tumor microenvironment is important for controlling epithelial–mesenchymal transition (EMT) in the pathogenesis, and progression of tumors, including prostate, bladder and brain cancers [58,61,62,63,64].

In this scenario, interfering with SHH pathway has been proposed as a promising strategy in GBM, even if co-players and mechanisms are largely unclear. Here we sought to investigate a relationship between CX43 and SHH signalling pathway in the complex GBM microenvironment.

Our results showed that two different human GBM cell lines actively express SHH signalling pathway effector SMO and we were able to modulate and interfere with canonical SHH pathway using purmorphamine and cyclopamine. In our study, cyclopamine on GBM cells was able to revert CX43 and GL1 expression induced by purmorphamine, but we did not observe a reduction of CX43 and GL1 expression compared to the control in cyclopamine exposed group. These effects were reflected in the functional experiments where we reported a significant increase of nuclear Ki-67 MFI and migration index in purmorphamine treated cells reverted by cyclopamine treatment. Previously studies demonstrated that cyclopamine interferes with GBM cell viability and stemness showing a synergistic effect with temozolomide [45,65]. However, in these studies, higher concentrations and long-time exposures of cyclopamine were used, whereas in our study we evaluated acute effects of both SMO stimulation and CX43 inhibition in order to find whether these treatments prelude to increasing migration and proliferation of GBM cell lines and may be reverted by cotreatments. SHH pathway inhibition by cyclopamine reduced clonogenicity in both GBM cell lines without affecting the metabolic turnover; it is worth noticing that MTT assay is not reflecting necessarily cell proliferation and growth, but viable cell metabolism [66]. Interestingly, we demonstrated that 1 µM purmorphamine was able to increase the clonogenicity of both GBM cell lines, while their surviving fraction was strikingly reduced with 10 µM. This similar result has been reported by our group on neuronal stems cells and it may be associated with a rebound effect and/or receptor desensitization [11]. Besides, confirming the modulatory function of SHH pathway on GBM cell machinery, we also found a potential relationship between purmorphamine exposition and CX43 levels. Such a link has been investigated in developmental studies and CX43 knock down models, in which a reduction of SHH levels and morphogens signalling activation have been observed upon CX43 inhibition and/or manipulation [33], thus suggesting a potential bidirectional interplay between CX43 and SHH signalling. Indeed, we found that even if a known SHH agonist (i.e., purmorphamine) induced a significant increase of CX43 levels, such an evidence was coupled with a loss of function in cotreatment with ioxynil octanoate, an inhibitor of CX43-based channels. Future studies are warranted to investigate CX43 phosphorylation and its channel-independent role, which may also affect cell machinery and have critical effects on proliferation and migration [67]. It is worth noticing that, in our experimental setting, inhibition of smoothened through cyclopamine does not affect the levels of CX43 nor the overall migration capabilities of GBM cell lines. This evidence supports the hypothesis that microenvironment in GBM may play a role in fostering GBM cells migration and proliferation, by stimulating SHH signalling and deregulating CX43 expression levels, thus modifying channels selectivity and microenvironmental conditioning [68].

## 5. Conclusions

Our results suggest a potential axis between CX43 and SHH signalling pathway at least on two main aspects: (i) “permissive”, similarly to developmental programs in which SHH-GLI signalling favors intercellular communication and patterning that leads to microenvironmental modification and disease/tumorigenic onset; (ii) “supporting” the stemness signature of GBM, so that aberrant SHH-GLI pathway, through modified CX43-mediated subsets cellular interaction, promotes cancer stem cell population critical for GBM self-renewal.

## Data Availability

The data presented in this study are available in the article or Supplementary Materials.

## Supplementary Materials

The following are available online at https://www.mdpi.com/article/10.3390/biology10080767/s1, Figure S1: Growth curves. Figure S2: Cytotoxicity assay. Figure S3: MTT turnover assay. Figure S4: Whole western blot showing all bands.
