# Supplementary material for: Connexin 43 and Sonic Hedgehog Pathway Interplay in Glioblastoma Cell Proliferation and Migration
Source: Biology (Basel). 2021 Aug 12;10(8):767. doi: 10.3390/biology10080767 (PMC8389699; doi:10.3390/biology10080767)
Supplement: Supplementary file 1 [file biology-10-00767-s001.zip › biology-1305849-supplementary.pdf]

## Supplementary Materials

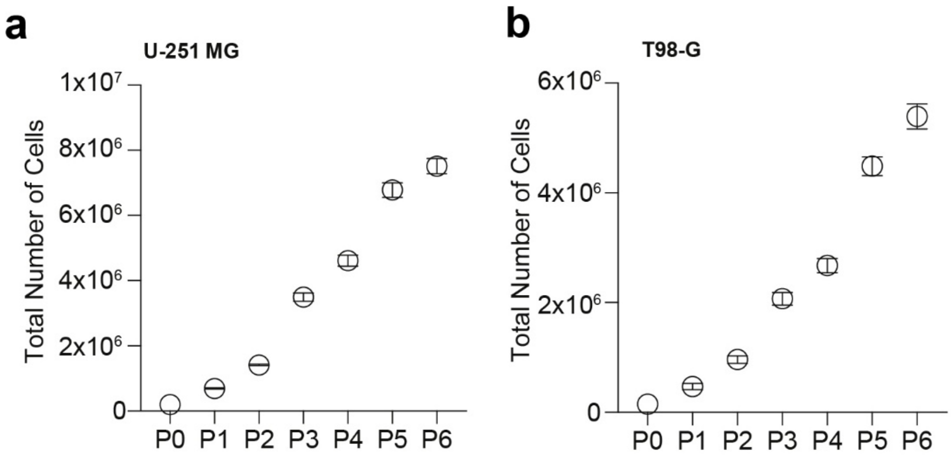


**Figure S1**. Growth curves. (**a**,**b**) Growth curve over six passages of human U251-MG (**a**) and T98-G (**b**) cell lines. Data are mean ± SEM of n = 3 independent cultures. P: passage.

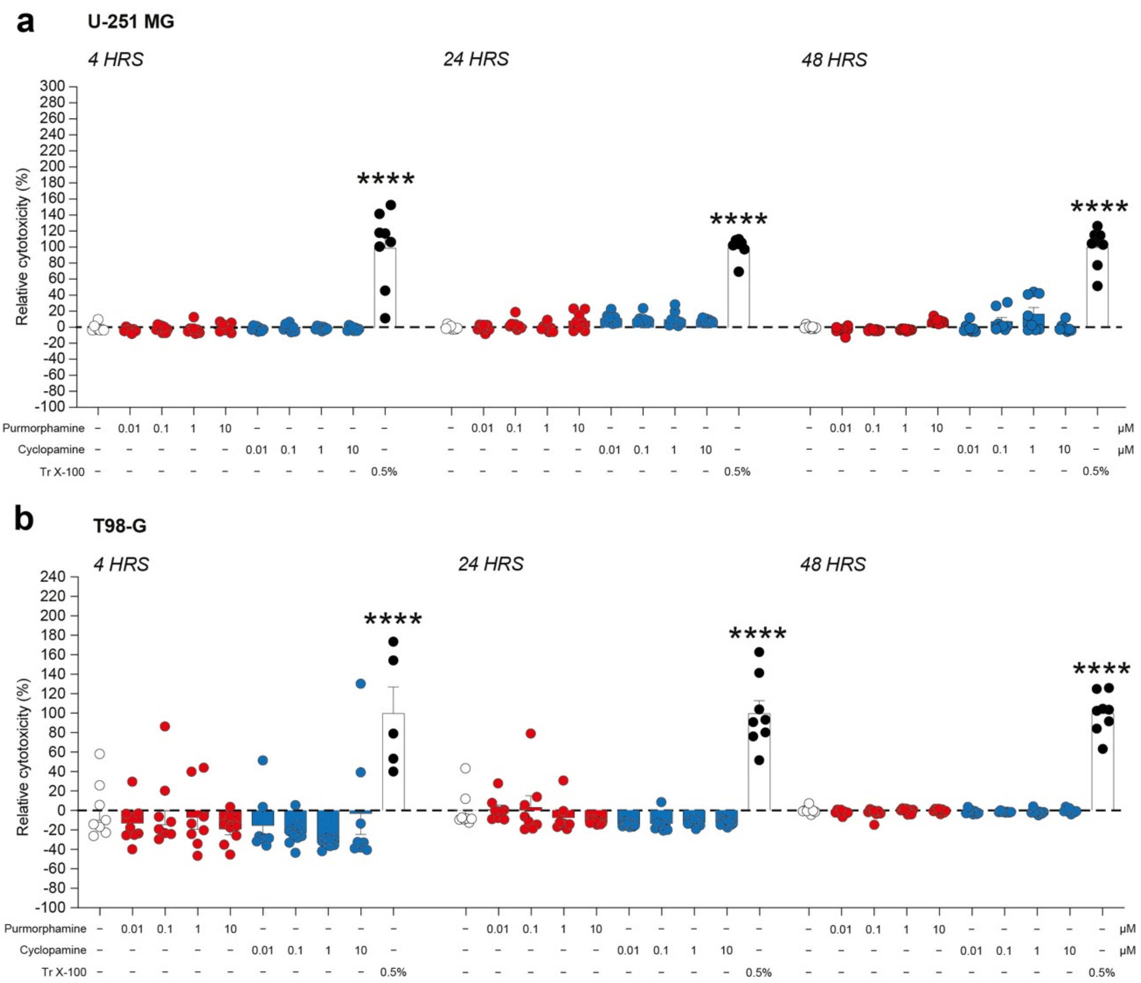


**Figure S2.** Cytotoxicity assay. (**a**,**b**) Cytotoxicity assay of positive and negative controls of human U251-MG (**a**), and T98-G (**b**) cell lines and cultures exposed to increasing concentration of purmorphamine and cyclopamine (0.01–10 μM). Data are mean ± SEM of n = 3 independent experiments; **** *p*-value < 0.0001 versus control; one-way ANOVA with Holm–Šídák post-hoc test. Tr X-100: Triton X-100.

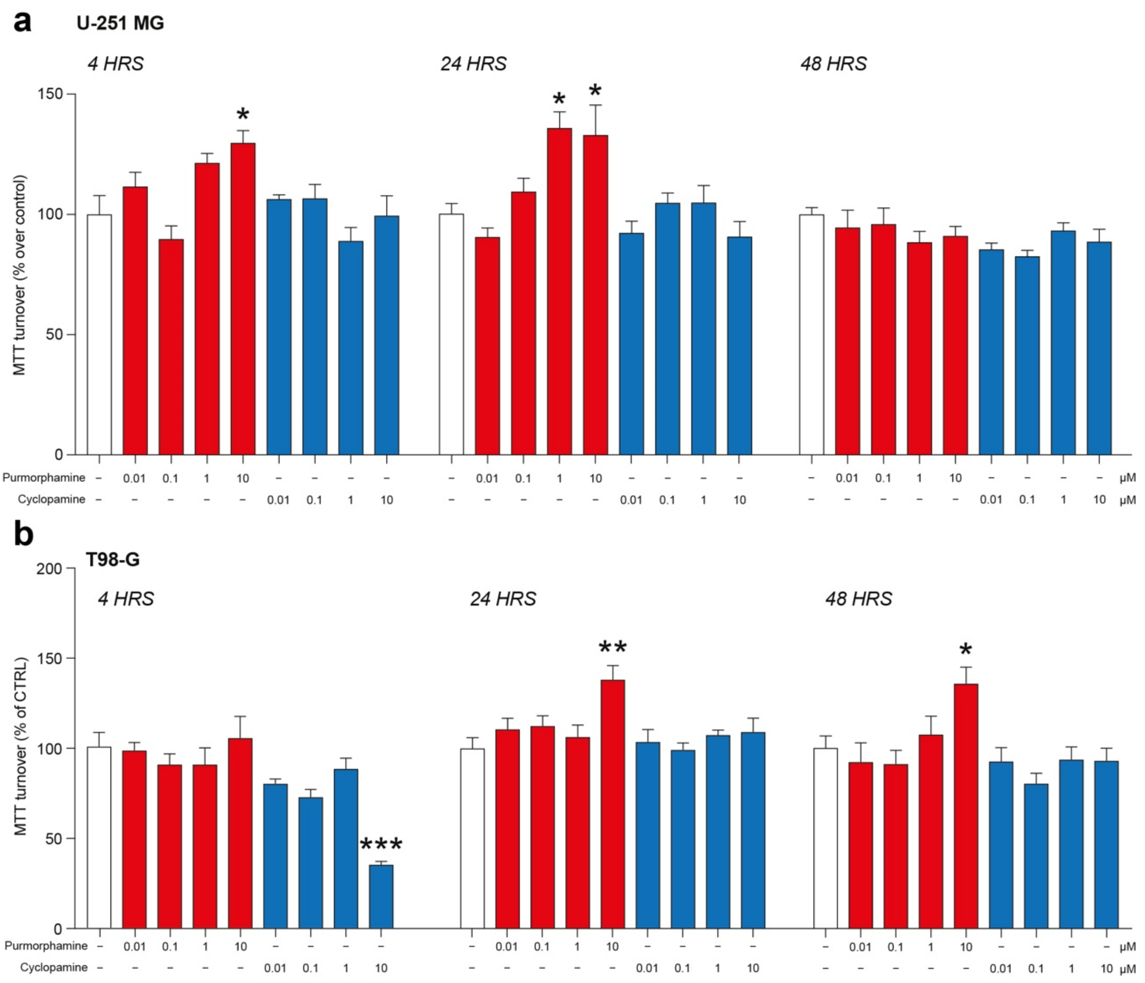


**Figure S3.** MTT turnover assay. (**a-b**) 3-(4,5-dimethylthiazol-2-Yl)-2,5-diphenyltetrazolium bromide (MTT) turnover of control human U251-MG (**a**) and T98-G (**b**) cell lines and cultures exposed to increasing concentration of purmorphamine and cyclopamine (0.01–10 μM). Data are mean ± SEM of n = 3 independent experiments; *p-value < 0.05, **p-value < 0.01 and ***p-value < 0.001 versus control; one-way ANOVA with Holm–Šídák post-hoc test.

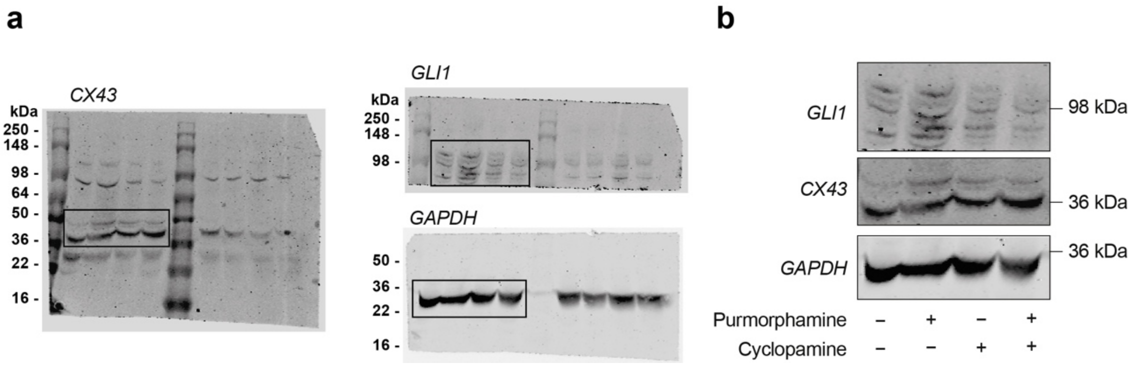


**Figure S4.** Whole Western blot showing all bands. (**a**) Uncropped membranes for CX43, GLI1 and GAPDH. (**b**) Cropped membranes showed in Figure 2c.
